# Influence of xanthine oxidoreductase inhibitor, topiroxostat, on body weight of diabetic obese mice

**DOI:** 10.1038/s41387-021-00155-2

**Published:** 2021-04-13

**Authors:** Takashi Nakamura, Mai Nampei, Takayo Murase, Etsuko Satoh, Seigo Akari, Noriaki Katoh, Hiroki Mizukami

**Affiliations:** 1 Pharmacological Study Group Pharmaceutical Research Laboratories, Sanwa Kagaku Kenkyusho, Mie, Japan; 2 Medical Affairs Department, Sanwa Kagaku Kenkyusho, Aichi, Japan; 3 Department of Pathology and Molecular Medicine, Hirosaki University Graduate School of Medicine, Aomori, Japan

**Keywords:** Biochemistry, Enzymes

## Abstract

Plasma xanthine oxidoreductase (XOR) activity is high in metabolic disorders such as diabetic mellitus, obesity, or overweight. Thus, this study investigated whether the XOR inhibitor, topiroxostat, affected body weight. Male db/db mice were fed standard diets with or without topiroxostat for 4 weeks. Body weight and food intake were constantly monitored, along with monitoring plasma biochemical markers, including insulin and XOR activity. Additionally, hepatic hypoxanthine and XOR activity were also documented. Single regression analysis was performed to determine the mechanism. Topiroxostat treatment suppressed weight gain relative to the vehicle without any impact on food intake. However, the weight of fat pads and hepatic and muscle triglyceride content did not change. Topiroxostat decreased the plasma uric acid and increased hepatic hypoxanthine in response to the inhibition of XOR activity. Plasma ketone body and free fatty acid were also increased. Moreover, fat weight was weakly associated with plasma XOR activity in the diabetic state and was negatively associated with ketone body by topiroxostat. These results suggested that topiroxostat amplified the burning of lipids and the salvage pathway, resulting in predisposing the body toward catabolism. The inhibition of plasma XOR activity may contribute to weight loss.

## Introduction

Xanthine oxidoreductase (XOR) is a rate-limiting enzyme that catalyzes purine metabolism of hypoxanthine to xanthine and xanthine to uric acid (UA). The activation induces hyperuricemia and gout in humans. Plasma XOR activity is high in individuals with metabolic syndrome or metabolic diseases such as diabetic mellitus, obesity, and overweight relative to healthy individuals. It is positively associated with body mass index, hemoglobin A1c (HbA1c), and insulin resistance^1–3^. Moreover, the plasma XO activity in obese children was higher than in those with healthy weight^4^, and weight loss by meal replacement therapy in obese patients decreased the plasma XO activity^5^. However, there have been few reports on weight change with an XOR inhibitor. Thus, in this study, we investigated whether the novel XOR inhibitor, topiroxostat, influences the weight in diabetic obese mice.

## Materials and methods

### Animal study

All experiments were approved by the Committee on Animal Care of Sanwa Kagaku Kenkyusho. Co., Ltd. Male littermate db/lean (db/m) and db/db mice obtained from Charles River Japan (Osaka, Japan) at 8 weeks of age were fed a standard powder diet. Non-diabetic mice (*n* = 14) and diabetic mice were divided into the control (*n* = 10) and topiroxostat (3 mg/kg/day; *n* = 10) groups matched for body mass, food intake, and HbA1c. Experiments were administered from 9 to 13 weeks of age. Mice were allowed ad libitum access to food and water. Body weight and food intake were measured constantly through the study.

### Blood and histological analysis

Blood samples were drawn from the inferior vena cava under 3% isoflurane, centrifuged to separate plasma, and kept at −80 °C until each assay. Plasma UA, free fatty acid (FFA), lactic acid (LA), triacylglycerol (TG), and ketone body were determined using an autoanalyzer (Hitachi, Tokyo, Japan). Plasma insulin was determined using an enzyme-linked immunosorbent assay kit (Shibayagi, Gunma). TG contents and adipocytes were assessed using a modified previously reported procedure^6^. In brief, homogenized tissues by a phosphate buffer were extracted with *a* chloroform-methanol mixture (2:1), and TG concentration was measured. A small piece of adipose tissue was fixed with 10% neutral-buffer formalin and was embedded in paraffin. The number of adipocytes was blindly counted under the microscope using hematoxylin and eosin staining. XOR activity was measured using [^13^C_2_,^15^N_2_]-xanthine as substrate, and the yielded [^13^C_2_,^15^N_2_]-UA was analyzed using liquid chromatography–triple quadrupole mass spectrometry (LC-TQMS)^7^. Hypoxanthine was similarly analyzed using LC-TQMS^7^.

### Statistical analysis

Data were expressed as mean ± standard error and were statistically analyzed by two-way repeated analysis of variance and *t*-test (Student’s or Welch’s test) using statistical analysis software (SAS version 8.0; SAS Institute, Cary, NC, USA). A *p* value of <0.05 was considered statistically significant.

## Results

### Physiological and biological data for topiroxostat treatment

**Fig. 1:**
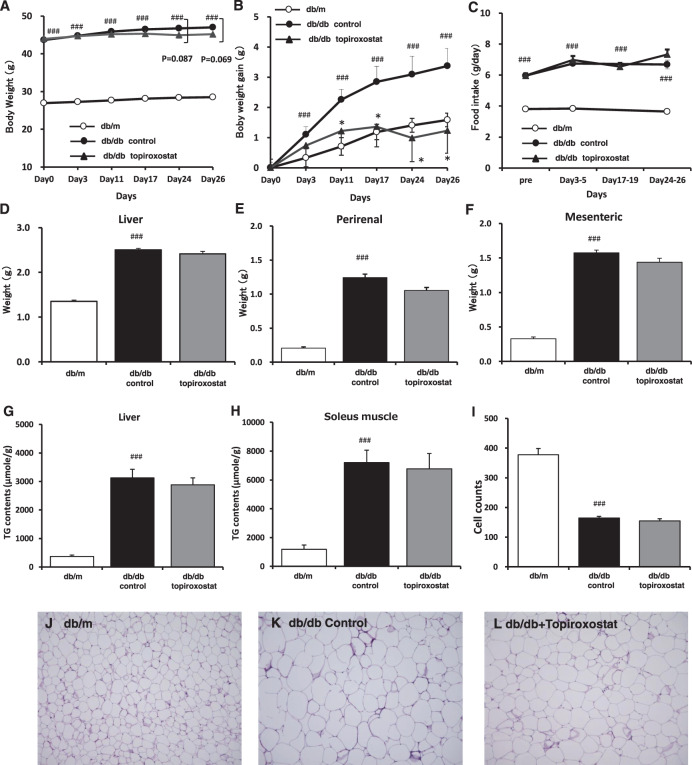
Physiological and histological data. Effects on body weight (**A**), changes in body weight (**B**), food intake (**C**), weight of liver (**D**), perirenal (**E**), mesenteric (**F**), TG contents of the liver (**G**) and soleus muscle (**H**), cell counts (**I**) and histology (**J**–**L**) of adipose tissues (db/m **J**; db/db control **K**; db/db + topiroxostat; **L**) during treatment with topiroxostat for 4 weeks. Data are mean ± standard error on db/m (*n* = 14), db/db control (*n* = 10), and db/db + topiroxostat (*n* = 10) in each group. ^###^*p* < 0.001 vs. db/m group (*t*-test), *p < 0.05 vs. db/db control group (*t*-test).

Body weight, weight gain, and food intake in the db/db group were higher than in the db/m group (Fig. 1A–C). The intervention of topiroxostat for 4 weeks suppressed weight gain relative to the db/db control without impact on food intake, but whole weight was not significant. Topiroxostat treatment did not alter the liver weight or the size of adipocytes compared with the diabetic control (Fig. 1D, I–L). However, the weight of the perirenal and mesenteric adipose tissue and the TG contents in the liver and muscles were likely to be slightly reduced (Fig. 1E–H).

**Fig. 2:**
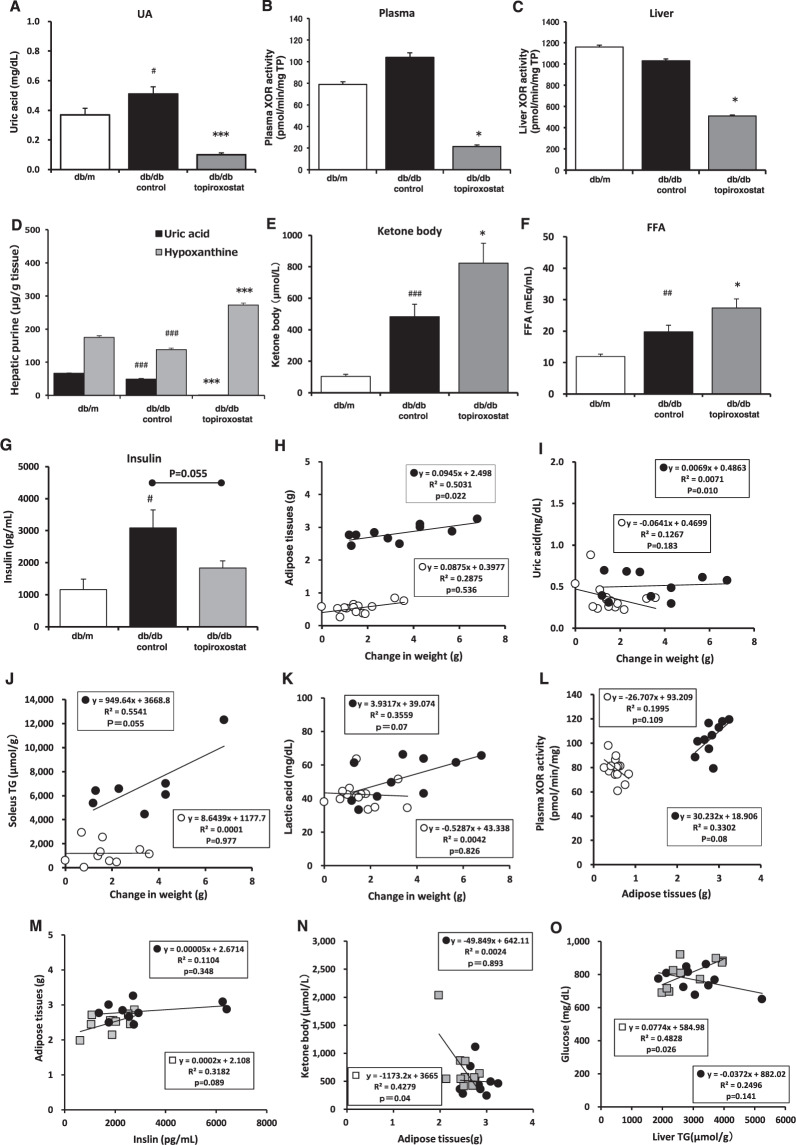
Biochemical data and single regression analysis. The measurement of plasma uric acid (**A**), plasma (**B**), and liver (**C**) XOR activity, hepatic purine bodies (**D**), ketone (**E**), FFA (**F**), and plasma insulin (**G**) during treatment with topiroxostat for 4 weeks. The relevance with the weight gain versus adipose tissues (**H**), uric acid (**I**), muscle TG contents (**J**), and lactic acid (**K**). Adipose tissues versus plasma XOR activity (**L**); plasma insulin versus fat weights (**M**); adipose tissues versus ketone bodies (**N**); and Hepatic TG contents versus plasma glucose (**O**) (thick outlined circle) in db/m (thin outlined circle), db/db control (solid circle), and topiroxostat treatment (open square). Data are mean ± standard error on db/m (*n* = 14), db/db control (*n* = 10), and db/db+topiroxostat (*n* = 10) in each group. ^#^, ^##^, ^###^ *p* < 0.05, <0.01, <0.001 vs. db/m group (*t*-test), *, *** *p* < 0.05, *p* < 0.001 vs. db/db control group (*t*-test).

Moreover, topiroxostat treatment decreased plasma UA and inhibited the plasma and liver XOR activity compared with the db/db control. Simultaneously, hypoxanthine increased, and UA decreased in the liver (Fig. 2A–D). Thus, the involvement of topiroxostat was confirmed, with a dose of 2.99 ± 0.1 mg/kg. The plasma ketone body, FFA, and insulin in the diabetic control were higher than in the db/m group. Ketone body and FFA levels were higher with topiroxostat compared with the db/db control, but insulin levels were reduced (Fig. 2E–G).

To determine why topiroxostat could suppress weight gain, we first analyzed the physiological and biological characteristics in the present diabetic mouse model. The result showed that weight gain was positively associated with the weight of adipose tissues, plasma UA, muscle TG contents, and plasma LA in the diabetic mice (Fig. 2H–K). Additionally, adipose tissues showed a weakly positive relevance with plasma XOR activity (Fig. 2L). These represented a typical obesity model with ectopic fat accumulation, and plasma XOR activity could contribute to the development of obesity. In topiroxostat treatment, plasma insulin was weakly associated with the adipose tissues (Fig. 2M), and the adipose tissues showed a negative relevance with the ketone body (Fig. 2N). Moreover, hepatic TG contents were correlated with plasma glucose (Fig. 2O).

## Discussion

This is the first time it has been reported that an XOR inhibitor, topiroxostat, has been shown to suppress body weight gain without impacting food intake. There have been some reports that body mass was slightly affected by treatment with the XOR inhibitors, allopurinol and febuxostat, using the obese model in rodents^8–13^. According to previous reports, treatment with allopurinol and febuxostat did not alter the body weight in an obese model induced by a high-fat diet^8^ or in a genetic obese model such as db/db^9,10^ or ob/ob^11^. However, allopurinol and febuxostat decreased or did not affect the weight in a metabolic syndrome model induced by a high-fructose diet, respectively^12,13^. It induced the mechanically obese model through enhancement of the XOR activity with the conversion of adenosine 5′-triphosphate (ATP) to adenosine 5′-diphosphate (ADP) by fructokinase in the liver^14^. Besides, XOR heterogenic mice showed weight gain relative to the homogenic mice^15^, and XOR null mice showed a 50% reduction in adipose mass compared with the wild type^16^. However, these XOR-specific genetic models had issues of lactation impairment and premature death within 4 weeks, respectively^17,18^. As described above, although allopurinol, febuxostat, and topiroxostat were all XOR inhibitors, these inhibitors were likely to act differently. It could be that the binding mode against the XOR enzyme^19^ and the intensity of inhibitory activity to plasma XOR among those drugs were different^7,20^. Thus, it was suggested that topiroxostat could suppress weight gain because it was the strongest inhibitor for plasma XOR^7,20^.

Insulin is one anabolism hormone secreted from the pancreas, which has glucose and fatty acid uptake or TG synthesis in the liver, muscle, and adipose tissues. Generally, TG was decomposed to glycerol and fatty acid, and the fatty acid was metabolized to ketone bodies via acetoacetyl-CoA from acetyl-CoA by β-oxidation in the liver. In this study, topiroxostat lowered the high insulin level in the db/db control and increased the plasma levels of ketone bodies and FFA (Fig. 2E–G). These changes were associated with the weight of fat (Fig. 2M, N). These results indicated that the systemic condition was closer to catabolism rather than anabolism. However, although the mass in adipose tissue and TG contents in the liver and muscle were not significantly decreased (Fig. 1E–H), it could be a result of short-term administration. In the viewpoint of the whole body, the percent body fat would be lower. Thus, it was suggested that topiroxostat could decrease adiposity by burning lipids or suppressing intake of lipids.

Inhibiting the XOR activity has the suppressive effect of UA and O_2_-induced reactive oxygen species production as well as the activation of salvage pathway by increasing hypoxanthine, which is also the substrate of hypoxanthine phosphoribosyl transferase (HPRT). In this study, topiroxostat increased the hepatic hypoxanthine level (Fig. 2D), suggesting that topiroxostat may induce HPRT activity and help supply nucleotides (ATP, ADP, etc.) as energy sources. The plasma LA level in diabetic obese mice was higher than in that of the db/m mice (52.3 ± 4.0 mg/dL vs. 42.5 ± 2.1 mg/dL; *p* < 0.05 vs. db/m), and hepatic TG content was positively associated with plasma glucose by topiroxostat treatment (Fig. 2O). This was considered a hypoxic state, and there was a shortage of ATP production for metabolic energy in the cell. Therefore, it was suggested that topiroxostat could improve the intracellular metabolic pathway followed by enabling to effectively yield ATP regardless of anaerobic condition. Moreover, according to the single regression analysis in the diabetic mice, fat weight showed a weak positive correlation with plasma XOR activity (Fig. 2L). Thus, it was suggested that elevated plasma XOR activity could contribute to the overweight condition. Therefore, topiroxostat intervention could be suitable for hyperuricemic obese or overweight patients.

In summary, topiroxostat suppressed body weight gain without affecting food intake. It may be because topiroxostat amplified the burning of lipids and glucose usage. This enhanced the salvage pathway by increasing hepatic hypoxanthine, resulting in the predisposition of the body toward catabolism. Moreover, the elevation of plasma XOR activity contributed to obesity in diabetic mice. Therefore, it is suggested that the inhibition of plasma XOR activity may contribute to weight loss.
